# Molecular factors in migraine

**DOI:** 10.18632/oncotarget.9367

**Published:** 2016-05-14

**Authors:** Marta Kowalska, Michał Prendecki, Wojciech Kozubski, Margarita Lianeri, Jolanta Dorszewska

**Affiliations:** ^1^ Laboratory of Neurobiology, Department of Neurology, Poznan University of Medical Sciences, Poznan, Poland; ^2^ Chair and Department of Neurology, Poznan University of Medical Sciences, Poznan, Poland

**Keywords:** gene polymorphisms, biochemical factors, migraine

## Abstract

Migraine is a common neurological disorder that affects 11% of adults worldwide. This disease most likely has a neurovascular origin. Migraine with aura (MA) and more common form - migraine without aura (MO) – are the two main clinical subtypes of disease. The exact pathomechanism of migraine is still unknown, but it is thought that both genetic and environmental factors are involved in this pathological process. The first genetic studies of migraine were focused on the rare subtype of MA: familial hemiplegic migraine (FHM). The genes analysed in familial and sporadic migraine are: *MTHFR*, *KCNK18*, *HCRTR1*, *SLC6A4*, *STX1A*, *GRIA1* and *GRIA3*. It is possible that migraine is a multifactorial disease with polygenic influence.

Recent studies have shown that the pathomechanisms of migraine involves both factors responsible for immune response and oxidative stress such as: cytokines, tyrosine metabolism, homocysteine; and factors associated with pain transmission and emotions e.g.: serotonin, hypocretin-1, calcitonin gene-related peptide, glutamate. The correlations between genetic variants of the *HCRTR1* gene, the polymorphism 5-HTTLPR and hypocretin-1, and serotonin were observed. It is known that serotonin inhibits the activity of hypocretin neurons and may affect the appearance of the aura during migraine attack.

The understanding of the molecular mechanisms of migraine, including genotype-phenotype correlations, may contribute to finding markers important for the diagnosis and treatment of this disease.

## INTRODUCTION

Migraine is classified as a primary headache disorder. The exact pathomechanism of migraine remains unclear, but it is believed that activation of the trigeminovascular system (TGVS) and cortical spreading depression (CSD) play an important role in this pathophysiological conditions. Clinically, migraine is divided into two main subtypes: migraine with aura (MA) and without aura (MO). CSD is manifested in the aura [1,2].

International Headache Society (IHS) criteria for migraine with and without aura from 2013 are presented in Table 1.

**Table 1:** International Classification of Headache Disorders 3rd Edition [3]

Migraine with aura (MA):	Migraine without aura (MO):
At least two attacks fulfilling criteria B and C One or more of the following fully reversible aura symptoms: visual sensory speech and/or language motor brainstem retinal At least two of the following four characteristics: at least one aura symptom spreads gradually over ≥5 minutes, and/or two or more symptoms occur in succession each individual aura symptom lasts 5-60 minutes at least one aura symptom is unilateral the aura is accompanied or followed within 60 minutes by headache Not better accounted for by another ICHD-3 diagnosis and transient ischaemic attack has been excluded.	At least five attacks fulfilling criteria B-D Headache attacks lasting 4-72 hours (untreated or unsuccessfully treated) Headache has at least two of the following four characteristics: unilateral location pulsating quality moderate or severe pain intensity aggravation by or causing avoidance of routine physical activity (e.g. walking or climbing stairs) During headache at least one of the following: nausea and/or vomiting photophobia and phonophobia Not better accounted for by another ICHD-3 diagnosis.

## EPIDEMIOLOGY AND RISK FACTORS

Migraine affects 11% of the adult population around the world [4]. Studies performed in the United States and Europe has shown that migraine has a three-time higher rate in women (15-18%) than in men (6-8%). The course of the disease may differ depending on gender, and it is related with ovarian hormones [5, 6]. Moreover, women experience more frequent, longer and stronger headaches and their headaches are more susceptible to develop into a chronic form [7]. Women are also more prone to photo- and phonophobia and nausea as compared to men [8].

In general, migraine has its onset during puberty; however, the majority of patients who suffer from this disease are aged 35 to 45 years [4]. Migraine attack can be induced by certain foods (aged cheeses, salty foods and processed foods), food additives, drinks – especially wine – stress, sensory stimuli such as bright lights or unusual smells and medication. Another risk factor for migraine is obesity, and applies particularly to young adults and women in general [9-11].

According to population-based twin studies, genetic and environmental factors have almost equal input in the development of migraine [12]. However, studies on twins rose together and apart showed that environmental factors are less important [13]. Population-based family studies showed that first-degree relatives of a proband with MA have an almost four-fold increased risk to suffer from MA, and first-degree relatives of a proband with MO have an approximately two-fold increased risk for MO, both in comparison to the general population [14].

## GENE POLYMORPHISMS AND MIGRAINE

The first genetic studies of migraine focused on a rare monogenic subtype of MA, namely familial hemiplegic migraine (FHM). In the pathogenesis of FHM type 1, 2 and 3 mutations in *CACNA1A*, *ATP1A2* and *SCN1A* genes were identified, respectively. *CACNA1A* encodes a subunit of the Cav2.1 (P/Q type) voltage-gated neuronal calcium channel expressed throughout the central nervous system (CNS). 21 mutations affecting the clinical course of migraine have been identified in this gene [15]. *ATP1A2* encodes a subunit of the sodium-potassium pump, and within this gene 30 mutations were found, most of which do not affect the course of the disease [16]. The last gene associated with FHM, *SCN1A*, encodes a subunit of a neuronal voltage-gated sodium (Nav1.1) channel [17].

Genetic studies in FHM indicated that ion transporter genes and the neurotransmitter pathway play a significant role in migraine pathogenesis [18,19]. It is likely that migraine is polygenic/oligogenic with a wide genetic heterogeneity [20]. Molecular genetic studies of migraine have investigated many polymorphisms possibly associated with MA and/or MO. In this disease the most often examined genes involved in regulation of homocysteine (Hcy), serotonin (5-HT), hipocretin-1, and glutamate, are presented in Table 2.

**Table 2:** Overview of selected gene studies in migraine in recent years

Gene and location/ biochemical marker	Gene variant	Biochemical marker	Population	Patients (MA+MO)	Controls	References
*MTHFR* 1p36.3/ homocysteine	rs1801133	- - - - - - -	Mongoloid Caucasian Caucasian Caucasian USA population Meta-analysis Caucasian	74 (22+52) 652 (465+187) 413 (187+226) 898 (898+0) 4577 (1275+1951) 2961 (2170+791) 267 (165+102)	261 270 1212 900 20424 3844 -	[21] [22] [23] [24] [25] [26] [27]
*KCNK18* 10q25/TRESK	F139WfsX24, F103F, L143L, Y163Y, S252S, P282P, I289I, T322T and A34V, A233V rs363314, rs1617136, rs963975	- -	Caucasian Caucasian	511 340 (228+112)	505 345	[28] [29]
*SLC6A4* 17q11.2/ serotonin	5-HTTLPR	- - Serotonin - -	Caucasian Caucasian Caucasian Caucasian Caucasian	52 (23+29) 212 (59+153) 64 (0+64) 144 (52+92) 253 (58+235)	80 - 42 105 244	[30] [31] [32] [33] [34]
*HCRTR1* 17q21.2/hypocretin-1	rs10914456, rs4949449 and rs2271933	-	Caucasian	384 (54+330)	259	[35]
*STX1A* 7q11.23/ syntaxin 1A	rs6951030, rs941298, rs4363087 rs6951030, rs941298, rs3793243 rs6951030, rs941298	- - -	Caucasian Caucasian Caucasian	188 (86+102) 188 (77+111) 567	210 287 720	[36] [37] [38]
*GRIA1* 5q33.2/glutamate	rs548294, rs2195450	- - -	Caucasian Caucasian Caucasian	244 (135+109) 452 (333+119) 186 (0+186)	260 454 312	[39] [40] [41]
*GRIA3* Xq24-q28/glutamate	rs3761555	- -	Caucasian Caucasian	244 (135+109) 452 (333+119)	260 454	[39] [40]

Abbreviations: **MA** - migraine with aura; **MO** - migraine without aura; **MTHFR** - methylenetetrahydrofolate reductase; **KCNK18** - potassium channel subfamily K member 18; **TRESK** - TWIK-related spinal cord potassium channel; **SLC6A4** - solute carrier family 6 (neurotransmitter transporter), member 4; **5-HTTLPR** - serotonin-transporter-linked polymorphic region; **HCRTR1** - hypocretin (orexin) receptor 1; **STX1A** - syntaxin 1A, **GRIA 1**,**3** - glutamate receptor, ionotropic, AMPA 1,3

Methylenetetrahydrofolate reductase (MTHFR) is a key enzyme in Hcy metabolism, and is encoded by the *MTHFR* gene. MTHFR is essential for Hcy conversion to methionine [42]. Hcy is a potentially toxic amino acid, which in excess causes remodelling of vascular tissue. The initiation and maintenance of a migraine episode may be caused by Hcy-related endothelial dysfunction, reducing oxygen flux into the brain [22]. The most common polymorphism of *MTHFR*, rs1801133 (C677T), is not unique to migraine, and is also involved in heart diseases, neural tube defects, stroke, high blood pressure, glaucoma and a few other conditions [2,26,43]. This polymorphism leads to the substitution of alanine for valine at position 222 within the catalytic domain of the enzyme. Consequently, this substitution changes the quaternary structure of the protein and reduces enzyme activity. The mean activity of MTHFR in individuals carrying the CT genotype is 65%, while in TT variant carriers it reaches only 30%, both in comparison to the CC genotype [27, 29, 44]. The presence of genetic change directly leads to elevated levels of Hcy in the bloodstream - a condition known as mild hyper-Hcy, which is a risk factor for migraine and cardiovascular diseases. According to numerous studies in Caucasian and Mongoloid populations, the TT genotype significantly increases risk for MA, whereas it does not influence the MO subtype [21, 22, 27]. Recent meta-analyses have confirmed this hypothesis [26, 45]. Additional dietary intake of B vitamins (B6, B9, B12) might be a remedy for increased Hcy. It has been proven that daily supplementation of B vitamins over a six month period reduces the headache intensity and decreases the level of Hcy in migraine patients [46].

The *KCNK18* (potassium channel subfamily K member 18) gene was identified by Lafreniere et al. [28] as a first causal typical migraine gene in a multigenerational family with MA. This gene encodes the two-pore domain potassium (K2P) channel TRESK. The most interesting variant is a frameshift mutation (F139WfsX24) that causes premature truncation of the TRESK protein in the first transmembrane region. The mutation results in a non-functional protein because of a dominant-negative downregulation of the wild type channel. Functional analysis revealed that neurons expressing mutant TRESK subunits have a lower current threshold for action potential initiation and a higher spike frequency in response to suprathreshold stimuli. The result of mutation is hyperexcitability of trigeminal nerve neurons and probably an increase in the susceptibility of migraine headache due to the trigeminal nociceptive pathway [47]. It is suggested that TRESK can be a potential target for the development of new analgesics [48]. Additional screening of the *KCNK18* gene mutation has identified other missense variants. The rs772633496 (A34V) variant also causes a reduction in channel activity, whereas the rs140325655 (C110R) variant was found to cause a complete loss of TRESK function. These variants' association with migraine is still unclear [49]. Analysis of three common single nucleotide polymorphisms (SNPs) in the *KCNK18* gene - rs1617136, rs363314 and rs963975 - showed that there is no correlation of these SNPs with migraine [29].

It is believed that the serotoninergic system plays an important role in the pathogenesis of migraine. 5-HT1B and 5-HT1D receptor agonists (triptans) are known to be effective in migraine pharmacotherapy, whereas the influence of numerous polymorphisms in 5-HT1A, 5-HT1B, 5-HT2A and 5-HT2C in this disease is still being investigated. Most of the current results deny that changes in the 5-HT receptor sequence increase the risk of migraine [50 ,51]. Also the genetic variants of the 5-HT transporter gene *SLC6A4* are being analyzed in migraine. Two functional polymorphisms in the *SLC6A4* gene were observed. The first of them is a variable number tandem repeats (VNTR) polymorphism, 17 bp long, situated in the second intron. The 2.10 and 2.12 variant are more frequent in migraine patients, whereas meta-analysis showed an association with migraine only of the 2.12 variant [30, 52, 53]. In contrast, the insertion/deletion polymorphism 5-HTTLPR (5-HT-transporter-linked polymorphic region) located in the regulatory region of this gene occurs in two allelic forms: the long variant (L) and the short variant (S), varying in length by 44 bp. The frequency of the short allele in the healthy population is 42-43% [54, 55]. It has been shown that the deletion (S) variant of HTTLPR leads to reduction of 5-HT transporter expression, which decreases the reuptake of 5-HT and can contribute depression [56]. Meta-analyses did not show a statistically significant relationship between the 5-HTTLPR polymorphism and migraine in the general population, as well as in family studies [56, 57]. However, Schürks et al. [57] indicated that European women carrying the S allele are twice as likely to develop migraine as European women without this allele.

*HCRTR1* (hypocretin receptor 1) is another candidate gene associated with migraine. The hypocretin (orexin) system involves the neuropeptide transmitters hypocretin-1 and hypocretin-2 (orexin-A and -B, respectively) and their G-protein coupled receptors (HCRTR1 and HCRTR2, respectively) [58]. Several genetic changes have been described in the *HCRTR1* gene. Non-synonymous polymorphism rs2271933 (G1222A) leads to the substitution of isoleucine by valine at position 408. This polymorphism is a risk factor of MO. Individuals, with the AA genotype have a two-fold increased risk of disease as compared to carriers of the GG genotype. It has been shown that the frequency of the A allele of rs2271933 varies among different populations. In the European population, allele A is found in 37%, while in Asian and African populations it achieves more than 70%. No connection to migraine was observed with the rs10914456 and rs4949449 polymorphisms [35, 59]. The hypocretin system controls several functions, for example: pain modulation, regulation of the autonomic system, and stress response. According to several studies, there is a relation between hypocretin-1 and the nociceptive phenomena and autonomic changes observed in primary headache disorders [60, 61]. Studies in the rat neuropathic pain model have shown that hypocretin-1 microinjection into the posterior hypothalamus causes an analgesic effect by decreasing the A- and C-fibre response to dural and electrical stimulation. The effect of hypocretin-1 on TGVS is also presynaptic, probably from decreased calcitonin gene-related peptide (CGRP) release, induced glutamate, and to a small extent GABA (γ-aminobutyric acid) release [62-64]. Activation of HCRTR1 inhibits neurogenic vasodilation, comparable to the action of sumatriptan. In migraine patients, it was also observed that CSD is inhibited by a dual hypocretin receptor antagonist, DORA-12. It has been suggested that targeting the hypocretin system may be used in novel migraine treatment [63, 65].

Glutamate is an excitatory neurotransmitter in the CNS and increases susceptibility to CSD and activates the TGVS. The *GRIA1-GRIA4* genes encode four types of subunits (GluR1-GluR4, respectively) of the AMPA receptor [66]. *GRIA1* and *GRIA3* (subunits GluR1 and GluR3) gene polymorphisms are considered potential predisposing/causative factors for migraine. The relationship between the *GRIA1* rs548294 and rs2195450 polymorphisms and MA (either as single markers or in haplotype combination) was demonstrated by Formicola et al. [39]. This group noted that the rs548294 variant was previously associated with MO. It has been suggested that some SNPs may be connected both with MA and MO as a result of a common pathomechanism of these subtypes [67]. The lack of association between *GRIA1* polymorphisms and haplotypes with MO or response to triptans was presented in other studies [40, 41]. However, an association was observed with the *GRIA3* polymorphism rs3761555 within the MA subgroup in two independent studies. Moreover, polymorphisms in the regulative regions (rs2195450 *GRIA1* and rs3761555 *GRIA3*) modify binding sites of promotors to transcription factors and decrease the expression of these genes [39, 40].

Another mechanism involved in the pathomechanism of migraine may include syntaxins, which are nervous system-specific proteins implicated in the docking of synaptic vesicles with the presynaptic membrane [68]. Syntaxin 1A, encoded by the *STX1A* gene, is involved in the regulation of neurotransmitters such as GABA by binding to the GABA transporter and inhibiting reuptake of GABA and 5-HT by decreasing the expression and changing subcellular localization of the 5-HT transporter [38]. Various SNPs were investigated to confirm the involvement of the *STX1A* gene in migraine susceptibility. The TT genotype of rs941298 showed a significant allelic association with MO. The haplotype analysis demonstrated involvement of the rs6951030 G allele in migraine susceptibility and a protective role for the T allele both for MA and migraine in general. SNP rs2293489 is also connected to migraine; however, no association was found between rs4363087 and rs3793243 variants and the disease [36-38]. All these studies support the role of the *STX1A* gene as a putative risk factor for migraine.

## BIOCHEMICAL MARKERS ASSOCIATED WITH MIGRAINE

According to the definition proposed in 1998 by the National Institutes of Health Biomarkers Definitions Working Group, a biomarker is “a characteristic that is objectively measured and evaluated as an indicator of normal biological processes, pathogenic processes, or pharmacologic responses to a therapeutic intervention”. Biomarkers can be divided into diagnostic, therapeutic, risk, progression, and/or prognostic indicators [69]. Characteristics of an ‘ideal’ biomarker:

- high sensitivity and specificity;
- high predictive value;
- analytical stability;
- relatively easy, cheap and low-invasive method of analysis;
- repeatability of method [70].

There are no validated biomarkers for migraine due to a lack of substance/genetic variants that are specific only to migraine or there are not enough studies on such biomarkers.

### Markers associated with inflammation and oxidative stress

According to several studies, proinflammatory cytokines such as IL-1 (interleukin-1) and IL-6 have been implicated in migraine. IL-1α is elevated in the blood of children with MA [71]. In adults with MA, the plasma levels of IL-1β are higher during headache-free time and in the early stage of attacks in comparison to MO patients [72]. Concentration of IL-6 is increased during the first 2 hours of migraine; moreover, IL-10 and tumour necrosis factor alpha (TNF-α) are also elevated during attacks. It is believed that other inflammatory markers associated with vascular dysfunction, e.g. Hcy and matrix metalloproteinase-9 (MMP-9), are elevated in the blood of migraine sufferers [73].

An elevated Hcy serum concentration has been linked to MA [74]. Also, the relationship between elevated Hcy levels and higher frequency and severity of migraine has been observed [46]. However, other studies did not support these results [75]. Furthermore, hyper-Hcy appears to cause MA attack initiation through changes in pain threshold [76].

### Markers associated with pain transmission and emotions

Biochemical studies have demonstrated metabolic abnormalities in the synthesis of neuromodulators and neurotransmitters in migraine, especially in MO. For instance, changes in the tyrosine metabolic pathway lead to an abnormal production of noradrenaline (NE) and dopamine (DA) with increased level of trace amines, such as tyramine, octopamine and synephrine. This results in impaired mitochondrial function and higher glutamate concentration in the CNS. The unbalanced levels of these neurotransmitters and neuromodulators (trace amines) in the synaptic dopaminergic and noradrenergic clefts of the pain pathways may activate the TGVS that releases CGRP. This phenomenon directly induces the migraine attack [77, 78].

CGRP is linked with pain transmission and inflammation promotion. CGRP is released in response to TGVS activation [79] and during severe migraine attacks [80]. Infusion of CGRP triggers migraine-like attacks in MA patients [81]. The saliva and plasma levels of CGRP are higher between attacks in migraine patients as compared to healthy controls [73]. Studies in cultured trigeminal neurons showed that migraine pharmacotherapies can both inhibit CGRP transcription and reduce CGRP release, whereas TNF-α can stimulate transcription of this peptide [82]. According to another study, an elevated saliva level of CGRP correlates with significantly improved response to rizatriptan and suggests that CGRP may also be useful as a therapeutic marker [83].

The pathways that involve both TGVS and CSD may be activated by glutamate. An increased concentration of glutamate was observed in the plasma, platelets and cerebrospinal fluid (CSF) from migraineurs, also in those with chronic migraine. According to several studies, glutamate intake may be increased in MA, while it was reduced in MO in comparison to the control group [84-86]. Reduction of plasma glutamate concentration may be used as a marker of positive response to prophylaxis in MO patients [87].

5-HT released from platelets to plasma may be involved in the pathomechanism of aura. Impairment of 5-HT stored in platelets during attacks was observed by Izzati-Zade [88]. It was shown that, between attacks in migraine patients, the 5-HT plasma level decreases and the concentration of the corresponding metabolite hydroxyindololeacetic acid (5-HIAA) increases. This condition reverses during attacks [89, 90]. It has been suggested that low concentration of 5-HT facilitates the activation of the trigeminovascular nociceptive pathway induced by CSD. These observations confirm the hypothesis that migraine is a syndrome of low serotonergic disposition [51, 91].

A significantly higher concentration of hypocretin-1 in CSF was observed in patients with chronic migraine and a correlation with painkillers intake was observed. The high hypocretin-1 level may be associated with early stages of migraine attack [65, 92, 93]. Another study performed on patients suffering from cluster headaches demonstrated reduced hypocretin-1 levels in CSF. This suggests that lower hypocretin-1 concentration may reflect insufficient antinociceptive activity of the hypothalamus [94].

The new targets for migraine therapy, such as CGRP receptor antagonist or anti-CGRP antibody, 5-HT1F agonist, glutamate antagonist and dual hypocretin-1 receptor antagonist, are currently in phase II clinical trials [95, 96].

## GENOTYPE-PHENOTYPE STUDIES IN MIGRAINE

There has only been a single genotype-phenotype correlation study in the case of migraine including the genetic and biochemical markers mentioned above. Juhasz et al. [32] demonstrated that the 5-HTTLPR SS genotype is associated with a higher 5-HT plasma level in the general population regardless of the occurrence of migraine.

Additionally, it has been shown that, under the physiological conditions, a high concentration of 5-HT reduces the pain threshold and increases pain intensity [97]. Subsequently, there is no firm conclusion on the impact of plasma hypocretin concentration in migraine. To date, there have been no studies analyzing hypocretin-1 level in plasma and its correlation with 5-HT in migraine patients.

In our unpublished preliminary data carried out on 34 Polish migraine patients (MA:17, MO:17) and 34 healthy controls, the correlations between 5-HT, hypocretin-1, 5-HTTLPR and rs2271933 polymorphism were analyzed. The presence of the 5-HTTLPR polymorphism was determined by polymerase chain reaction (PCR) and visualized by electrophoresis on 2% agarose gel, and the rs2271933 polymorphism of *HCRTR1* was analyzed using the high resolution melting analysis (HRMA) method with sequencing. The plasma concentration of 5-HT was estimated using the high performance liquid chromatography system with electrochemical detection (HPLC/EC), while the concentration of hypocretin-1 was estimated using ELISA kit. Our study has shown that the frequency of the L allele in migraine patients, both MA and MO, was higher than in the control group, and the SS genotype was more frequent in the MO group in comparison to the control group (Kruskal-Wallis test, p<0.05). Moreover, the L allele of 5-HTTLPR was associated with a tendency to increased 5-HT level, which did not correspond to the results demonstrated by Juhasz et al. [32]. However, it did correlate with the *in vitro* functional studies by Heils et al. [98], which showed a 2-fold higher expression of the 5-HT transporter in the L variant. As was previously mentioned, the function of the 5-HT transporter determines the 5-HT plasma level.

Additionally, it was observed that the 5-HTTLPR SL genotype leads to a significant reduction of hypocretin-1 plasma level (Kruskal-Wallis test, p<0.05) in migraine patients in comparison with controls. What is more, the S allele of the 5-HTTLPR polymorphism was correlated with a tendency to decreased plasma 5-HT levels in the general population. Furthermore, it was shown that the rs2271933 polymorphism of the *HCRTR1* gene probably does not change the plasma concentration of hypocretin-1; however, the allele A of this polymorphism was more commonly observed in migraine patients (control: 28%, migraine: 47%; Fisher's exact test, p<0.05). The hypocretin-1 plasma concentrations differ significantly between migraine patients and the control group (0,910±0,295 and 1.202±0.323ng/ml, respectively; Mann-Whitney test, p<0.05). There was no correlation between 5-HT and hypocretin-1 plasma levels in any group. To our knowledge, these yet unpublished data are the first genotype-phenotype studies among migraine in Poland.

**Figure 1:**
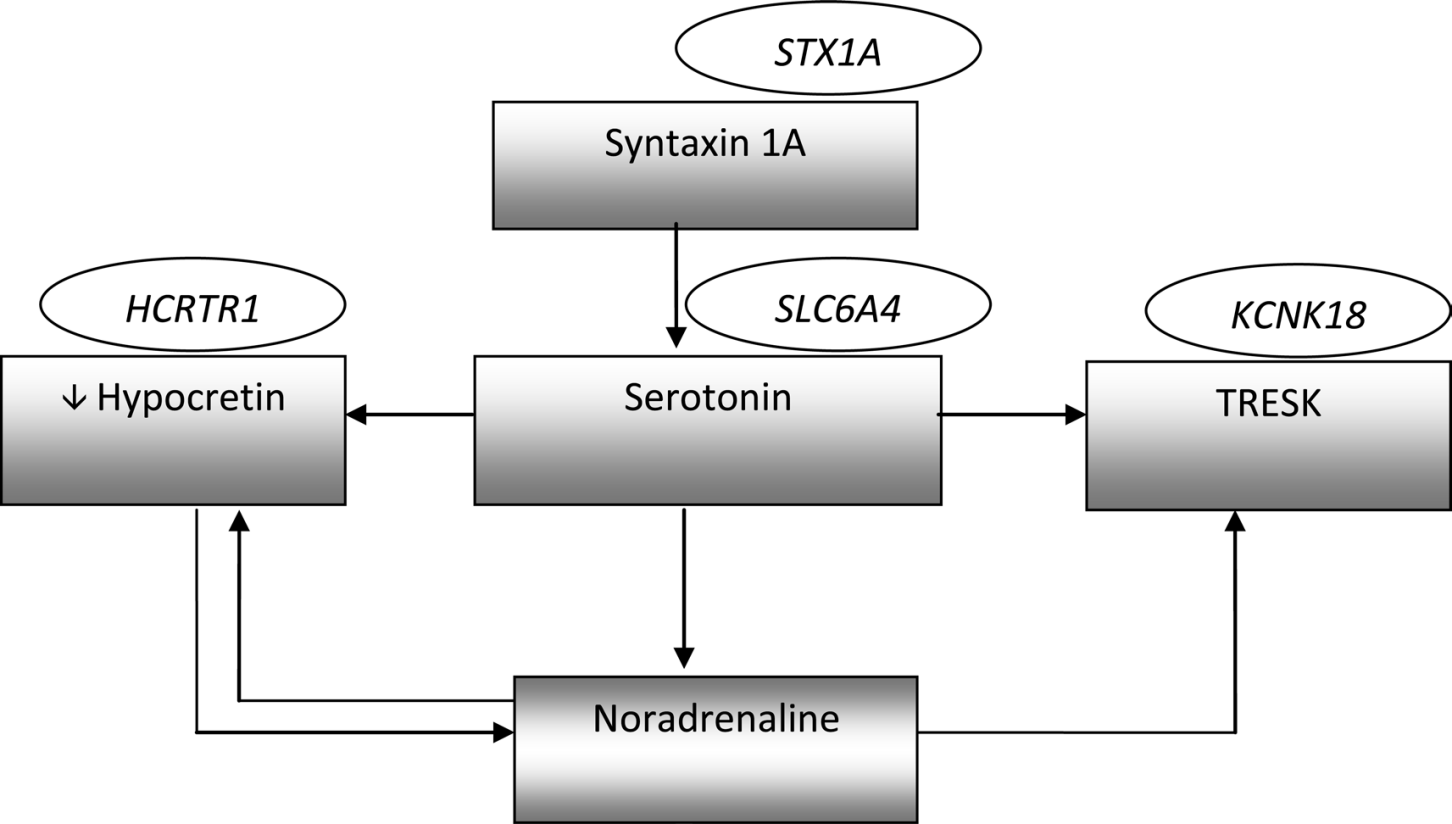
Interactions between mentioned molecules involved in migraine pathomechanism The genes names are shown in ellipses, while biochemical factors are shown in rectangles.

It seems that other factors associated with pain transduction, and generally emotions, may take part in the regulation of 5-HT and hypocretin levels (Figure 1).

It has been shown that 5-HT and NE inhibit the activity of hypocretin neurons via α_2_-adrenergic and 5-HT1A receptors. On the other hand, by the controlling noradrenergic transmission, hypocretin-1 indirectly changes the level of 5-HT. 5-HT1A receptors also cause the opening of potassium (K^+^) channels, leading to an efflux of K^+^ from the cell and hyperpolarization of the cell membrane [99]. Furthermore, both 5-HT and NE may inhibit the activity of TRESK channels by blocking the flow of K^+^ ions and increasing the excitability of neurons [100]. As was mentioned above, syntaxin 1A also regulates serotoninergic transmission via decreasing expression and changing localization of 5-HT transporter (Table 2., Figure 1).

## SUMMARY

Currently, diagnosis of migraine is based on questionnaires, interviewing and neuroimaging. Migraine is a result of the interaction between multiple genes with environmental factors and triggers. The discovery of genes involved in this disease may lead to new insights into the molecular pathways in the pathogenesis of migraine. The discussed polymorphisms may influence the phenotypic features of migraine patients, and have implications for the function of proteins. The analyzed markers were classified into two groups; those associated with immune and oxidative stress response: *MTHFR*, *GRIA1*, *GRIA3*, cytokines, and tyrosine metabolism, and those associated with pain transmission and emotional states: *KCNK18*, *HCRTR1*, *SLC6A4*, *STX1A*, CGRP, glutamate, 5-HT, and hypocretin-1. It appears that such biomarkers could be detected in the blood or saliva sample of individuals using biochemical or molecular methods and would be helpful in migraine diagnosis. These may also help in monitoring drug response, disease prognosis, and/or progression. Some of these mentioned molecular factors are currently under investigation as new targets for migraine pharmacotherapy. Moreover, the neuropeptides and their receptors may be used in the future treatment and prophylaxis of migraine.
